# Savanna burning methodology for fire management and emissions reduction: a critical review of influencing factors

**DOI:** 10.1186/s13021-016-0067-4

**Published:** 2016-11-16

**Authors:** Tek Narayan Maraseni, Kathryn Reardon-Smith, Greg Griffiths, Armando Apan

**Affiliations:** 1 Institute for Agriculture and the Environment, University of Southern Queensland, Toowoomba, 4350 Australia; 2 Natural Resources Management and Parks, South Burnett Regional Council, Queensland, 4610 Australia

**Keywords:** Kyoto Protocol, Emissions reduction funds, Carbon accounting, Savanna burning

## Abstract

Savanna fire is a major source of global greenhouse gas (GHG) emissions. In Australia, savanna fire contributes about 3% of annual GHG emissions reportable to the Kyoto Protocol. In order to reduce GHG emissions from savanna burning, the Australian government has developed and approved a Kyoto compliant savanna controlled burning methodology—the first legal instrument of this kind at a global level—under its Emission Reduction Fund. However, this approved methodology is currently only applicable to nine vegetation fuel types across northern parts of Australia in areas which receive on average over 600 mm rainfall annually, covering only 15.4% of the total land area in Australia. Savanna ecosystems extend across a large proportion of mainland Australia. This paper provides a critical review of ten key factors that need to be considered in developing a savanna burning methodology applicable to the other parts of Australia. It will also inform discussion in other countries intent on developing similar emissions reduction strategies.

## Background

Savannas, tropical and sub-tropical vegetation formations with continuous grass cover and occasional trees and shrubs [1], extend over more than 16% of the world’s land surface and contribute some 30% of total land-based net primary production [2]. The savanna biome is maintained by fire but, at the same time, fire is also a major source of global greenhouse gas (GHG) emissions. In tropical and subtropical regions (38°N–38°S), fire alone is responsible for emissions of about 4 PgC/year, the equivalent of ~10% of net primary productivity (NPP) in these areas [3]. About 50% of these emissions are attributable to Africa, 15–27% to South America and about 10% to Australia [3, 4]. Projected climate change is likely to further escalate fire related emissions from savannas, due mainly to changes in weather related attributes (temperature, rainfall, relative humidity, wind speed and solar radiation) and fuel related attributes (fuel load and moisture content) [5, 6].

In Australia, tropical savannas cover an area of about 2 million km^2^, over a quarter of the Australian continent [7], and represent about 12% of the world extent of tropical savanna ecosystems [8]. Subtropical savannas are also patchy in their distribution; hence, their overall extent is even greater. Savanna fire is one of the major contributors of national GHG emissions in Australia, accounting about 3% of annual emissions reportable under the Kyoto Protocol [9]. At a global level, Australia is ranked third for the amount of GHG emissions from savanna fire [10]. Hence, reducing emissions from savanna burning is of national interest in Australia. In addition to fire, biomass carbon in savanna systems is recycled through grazing and microbial decomposition.

Cattle grazing is one of the major land uses in savanna regions [8]; however, the role of grazing in fire management is a debatable topic. While grazing may decrease some of the combustible herbs and grasses, it also promotes woody shrubs and scrubs which are prone to crown fires [11, 12]. Hence, the role of grazing in fire management is of uncertain efficacy and is not an eligible activity under the current methodology [13].

Decomposition of leaf litter by microorganisms results in both lower GHG emissions and release over a longer period of time than does burning [14]. Complex mutualistic mechanisms among soil microorganisms further aid reduction in GHG emissions. For example, CH_4_ released by subterranean termites during the digestion of plant material is re-absorbed by bacteria in the soil [14]. Therefore, developing a strategy which reduces fire frequency and better harnesses these processes would result in more litter being decomposed/consumed via the microbial pathway and a reduction in GHG emissions [15]. Early dry season (EDS) burning or strategic prescribed burning has been found to be most effective in this regard as late dry season (LDS) fires emit 52% more emissions per unit area than do EDS fires [16].

Realising the benefits of EDS burning, the Australian government has developed and approved a Kyoto compliant savanna burning methodology under its emissions reduction funds (ERFs), allowing farmers and land managers to earn carbon credits by reducing methane and nitrous oxide emissions on the land [13, 17]. These credits can then be sold to the Australian Government, by participating in a reverse auction, or to people and businesses wishing to offset their emissions. In fact, this is the first legal instrument of this kind globally. The Paris Agreement, backed by 195 countries, has committed (non-bindingly) to assist poor developing countries for their mitigation and adaptation efforts with financial support of US$100 billion per annum by 2020 [18]. In this context, many savanna fire prone developing countries in Africa and South America, which collectively account for about 65–77% of total savanna burning emissions, may want to develop similar methodologies in order to receive carbon benefits.

**Fig. 1:**
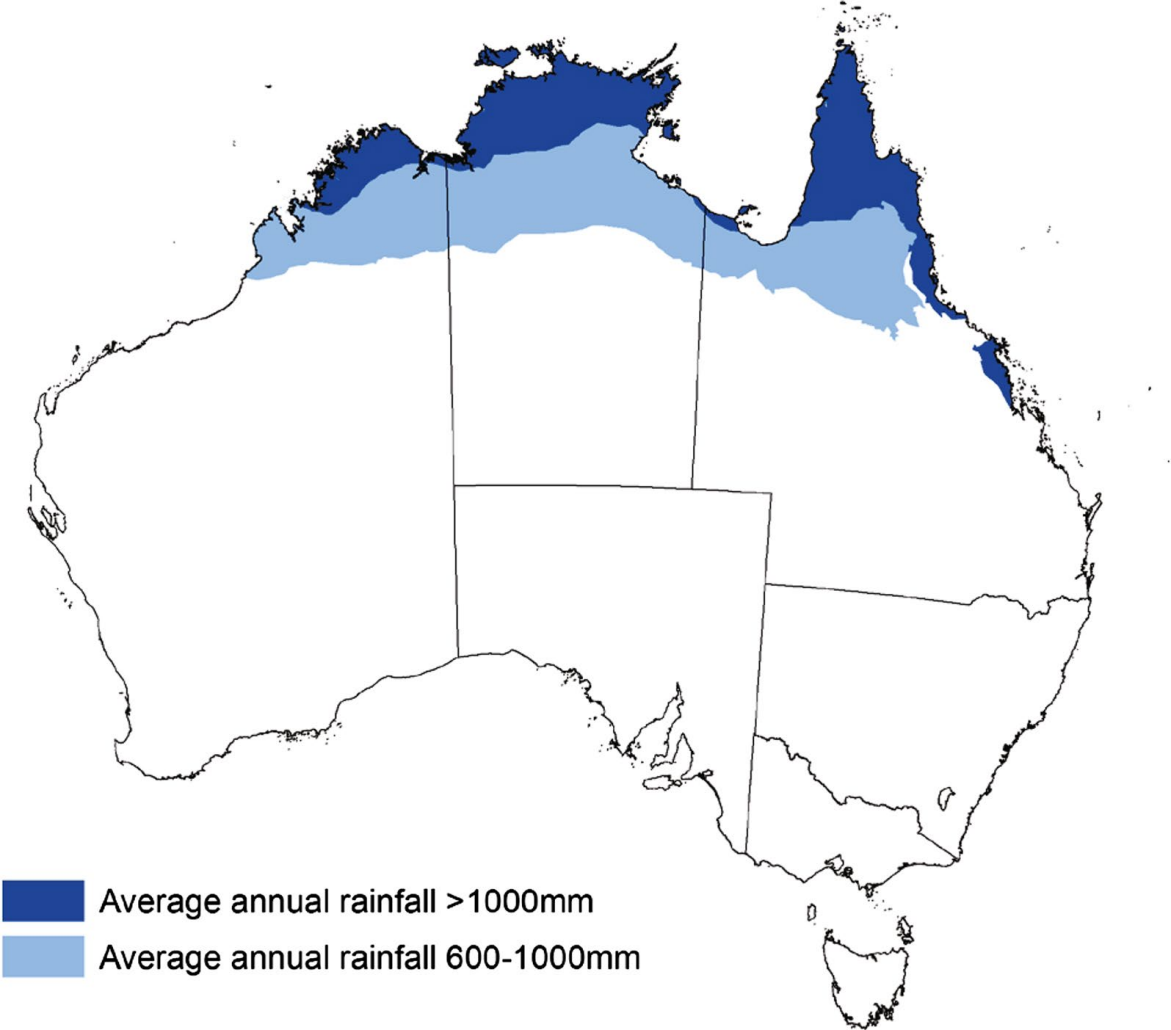
Rainfall map of Australia (area for 1000 mm annual rainfall = 472,326 sq km; area for 600–1000 mm average annual rainfall = 711,765 sq km)

The current savanna burning methodology in Australia applies only to areas of northern Australia which receive, on average, more than 600 mm of rainfall annually (Fig. 1). The methodology is separated into two different parts and covers two rainfall zones: (1) low annual average rainfall zone (600–1000 mm) with a land area of 472,326 sq km; and (2) high annual average rainfall zone (>1000 mm) with land area of 711,765 sq km [11, 15]. As such, the methodology only applies to about 59% of the 2 million km^2^ area of tropical savannas in Australia and 15.4% of Australia’s total land area (7,692,000 sq km). Many patches of sub-tropical and temperate grasslands, savannas and shrublands in Australia are not covered under this methodology. Moreover, the current methodology is applicable to only nine vegetation fuel types and cannot be applied to other vegetation fuel types in Australia. This study identifies those factors that need to be considered to enable a savanna burning methodology to be developed for southern parts of Australia (i.e. those parts of Australia where the current ‘northern Australia’ savanna burning methodology does not apply), in general, and sub-tropical and temperate grasslands, savannas and shrublands, in particular, which exhibit different vegetation fuel types and are subject to different climatic conditions. These aspects constitute the bulk of our paper’s contribution towards greater understanding of the issues relevant to developing a comprehensive savanna burning methodology.

The developed Australian Government methodology for northern Australian savannas provides some insights for African and South American countries wishing to develop similar savanna burning methodologies. This review, which critically discusses several critical factors for broader application, could provide new insights to aid their understanding and application. In this paper, we briefly discuss the magnitudes of GHG emissions from Australian savanna burning and then provide a brief snapshot of the current Australian Government savanna burning methodology. Finally, we review the ‘savanna burning’ related literature (journal papers, reports, book chapters) from Australia and elsewhere, where possible, and identify critical factors for extension of the savanna burning methodology to other parts of the world. While searching for relevant literature from online databases and websites, we used the following key words: “savanna”, “fire”, “burning” and “emissions”.

### GHG emissions from savanna burning in Australia

**Fig. 2:**
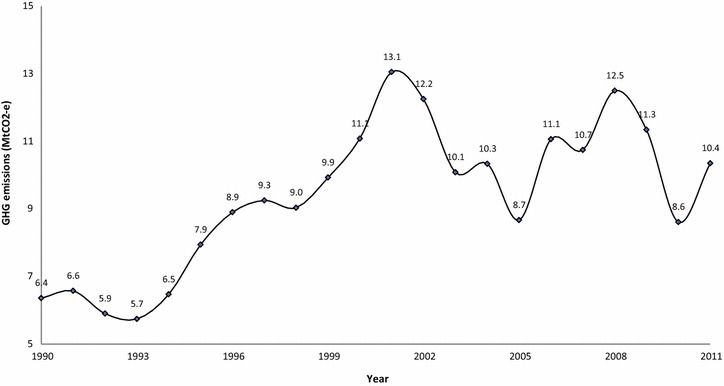
Greenhouse gas emissions (M t CO_2_e) from savannas burning in Australia (1990–2011) (adapted from Department of Environment [19]

Emissions from savanna burning are strongly related to patterns of rainfall and drought. For example, emissions from savanna burning in Australia increased by about 77% between 1990 and 2009 when the region experienced severe prolonged drought (the ‘millennium drought’), then fell to their lowest level in more than a decade following unusually high rainfall in 2010 (Fig. 2). Even so, savanna burning in 2010 still accounted for 8.6 Mt CO_2_e, equivalent to 10.9% of agricultural emissions in Australia [14]. Reducing emissions from savanna burning is of strategic importance in achieving the Australian Government’s commitment to reducing its GHG emissions.

### Emissions Reduction Fund and savanna burning

The Australian government’s Emissions Reduction Fund (ERF) is a voluntary scheme that allows farmers and other land managers to earn carbon credits by storing carbon or reducing GHG emissions on land. This would allow them to adopt a range of recommended ‘best’ management practices, and earn carbon credits and reputational benefits at the same time. As of 31 August 2016, there were 631 projects registered and 26.9 million Australian Carbon Credit Units (ACCUs) had been issued [20]. Of the 631 registered projects, 69 have used the savanna burning method, including: 19 in the Northern Territory; 38 in Queensland; 10 in Western Australia; and 2 multi-state jurisdictions [20].

Australia has set its new emissions reduction target of 26% below 2005 levels by 2030. Part of this target would be achieved from the ERF and part from other proposed safeguard mechanisms. Using the ERF, the clean energy regulator (CER) awards carbon abatement contracts following a process called a reverse auction—which allows it to choose the lowest priced abatement on offer. As of 31 August 2016, the CER conducted three auctions and awarded 309 Carbon Abatement Contracts to the value of $1.733 million. The successful contractors have committed to deliver 143 million tonnes of abatement of which 8 million ton of abatement will come from savanna burning. Therefore, savanna burning is becoming a vital component of the ERF [21].

### The Australian Government’s approved savanna burning methodology for northern Australia

In the savanna burning methodology approved for northern Australia, the estimation of potential emissions is based on area burnt, fire scar area, burning efficiency, fuel loads, emissions factors, carbon content and nitrogen to carbon ratio. Most of these values are derived from more than 15 years of experimental research at different times of the year in fire-prone tropical Australia (north of the Tropic of Capricorn). In this methodology, Australian carbon credit units (ACCUs) are calculated by subtracting the annual emissions from a baseline level derived from an average of the last 10 years’ (in the case of the higher rainfall zone) and 15 years’ (in the case of the lower rainfall zone) emissions prior to the commencement of the project [17]. If strategic fire management has been implemented in a project area for a period of 1–6 years before project commencement, the average emissions of the 10 and 15 years preceding this period is used for the estimation of baseline emissions. The 10 and 15 year baseline periods are used as these periods can be expected to cover one or more fire cycles and provide a reasonably reliable estimate of average emissions [16, 22].

The Australian Government’s approved savanna burning methodology is a Kyoto compliant methodology. In line with IPCC recommendations, it only considers CH_4_ and N_2_O emissions released by fire and does not include either carbon emissions from fires or carbon sequestration in soil or biomass as these are biogenic in nature [23]. This methodology is essentially aimed at: (1) reducing the area that is burnt each year; and/or (2) shifting the seasonality of burning from the late dry season (LDS) towards the early dry season (EDS) [13]. Using this methodology, the calculation of annual emissions involves several steps (see Australian Government [13] for details of the steps and sub-steps used in calculating annual emissions).

### Critical factors for consideration in developing a new savanna burning methodology

#### Incorporating both carbon sequestration and GHG emissions

In the Carbon Farming Initiatives legislation—now referred as the ERF—there is a provision for two types of Australian carbon credit units (ACCUs): (1) Kyoto-compliant ACCUs which can be sold into national and international compliance markets; and (2) non-Kyoto ACCUs which can be sold into volunteer carbon markets. Both types of carbon credits can be bought by the Australian Government through its emissions reduction funds [24]. The Australian Government has allocated about $4.95 billion dollars for the ERF by which it intends to meet part of its emissions reduction target of 26–28% of 2005 levels by 2030 [25]. The current methodology for savanna burring only considers the first type of ACCUs (in this case, reducing CH_4_ and N_2_O emissions from soils) and does not consider the second type (in this case, soil and biomass carbon sequestration activities).

By conducting prescribed savanna burning (PSB) or early dry season (EDS) savanna burning, some authors have suggested that large amounts of carbon could be sequestered in soils [26–28] and biomass [27, 29]. Others have suggested that if there is limitation in water availability, then potential increases in tree biomass are relatively limited, especially in relation to the dominant eucalypt component [29].

A number of approaches have been used to predict the long-term effects of fire on carbon stocks. For example, Cook et al. [30] applied both a statistical model, to assess carbon stock changes in live trees for up to 100 years, and the Flames model, to predict the effect of changing fire regime on carbon stocks in live trees, coarse woody debris and fine fuel. They concluded that the Flames process-based modelling approach provided a better estimation than did the statistical extrapolation approach.

There are however currently insufficient long-term experiments to support the contention that large amounts of carbon could be sequestered in soils and biomass; therefore, soil and biomass carbon sequestration are not accounted for in the current Australian Government savanna burning methodology which only assesses CH_4_ and N_2_O dynamics. For example, Richards et al. [27] report finding no measurable difference in soil C after 5 years of annual, 3 year and unburned fire treatments in mesic savanna of the Northern Territory. However, with the help of Century modelling they suggest that a detectable change in soil C (4 t/ha) might be achieved over 100 years. Modelled results also indicate that, over a 100 year period, soil C sequestration associated with reduced fire frequency and intensity in mesic savannas could be on average up to four times greater than reductions in non-CO_2_ emissions (i.e., CH_4_ and N_2_O) [27]. However, these results, and the reliability of the predicted soil C value after 100 years, are dependent on the values used for the parameterisation of the Century model and should perhaps be treated with a degree of caution.

Similarly, a study on the Tiwi islands north of Darwin predicted that about 89,700 tCO_2_-e (i.e., about 0.22 tCO_2_e/ha across an area of 4062 km^2^) could be sequestered in soil and vegetation carbon pools each year for 100 years [28]. However, eucalypts across northern Australia may be relatively insensitive to fire and their biomass largely controlled by water availability rather than by the frequency and intensity of fires [29]. On the other hand, reduction of fire frequency and intensity might be beneficial in increasing the biomass of fire-sensitive broadleaved and deciduous species [29]. Hence, the net carbon sequestration outcome at the landscape level is far from predictable.

Furthermore, a study by Livesley et al. [26] in the Northern Territory concluded that greenhouse gas (GHG) exchange between the atmosphere and savanna soils is dominated by CO_2_ flux, with soil-atmosphere CH_4_ and N_2_O exchange rates several times smaller in magnitude. However, soil carbon sequestration benefits could vary by type of vegetation and frequency of prescribed burning. For example, in the case of wet sclerophyll forests in southeast Queensland, soil carbon benefits, compared to baseline emissions, may be increased by conducting prescribed burning every 4 years [31]. This is possible as this area is likely to have, on average, one to two fire cycles every 15 years [17]. However, the outcome could be different in other vegetation types and under other climatic conditions.

Williams et al. [32] reported the impact of fire regimes on carbon stocks in terrestrial ecosystems in two contrasting biomes: (1) the tropical savannas of northern Australia, and (2) the temperate eucalypt forests of south–eastern Australia. They suggested that the potential carbon benefits of managing fire regimes in the savannas are greater than in temperate forests. Similarly, from a study using the eddy covariance method, Beringer et al. [33] reported that the net ecosystem productivity (NEP) of mesic savanna under a 5 year fire interval period was in the range of 3.5–5 tC/ha/year. Using the FLAME simulation model, Liedloff and Cook [cited in 32] suggested that the savannas were carbon sinks in 60–85% of years and that a higher percentage of sink years occurred with fire frequencies of 1 in 10 years.

There is limited study of fire impacts in southern eucalypt woodlands. A study by Kilinc et al. [34] reported that Mountain Ash forests of the Central Highlands of Victoria were a sink of 3.7 tC/ha/year. Unplanned fire intervals in south–eastern Australia are relatively long at around 20–25 years [35, 36]. Temperate eucalypt forests may act as net carbon sinks over a given return period of unplanned fires as the recovery time of NEP is much lower [32, 34] than 20–25 years. However, how these forests behave in terms of NEP with a changing climate is yet to be understood and there is therefore a need for research in this area.

Savanna fires are also a major source of black carbon aerosols. While how much exposed carbon could be converted into black carbon is uncertain, one estimate shows that it could be less than 3% [2]. However, there have been very few experimental studies. An empirical study in South Africa reported that the conversion could be in the range of 0.6–1.5% [37]. Forbes et al. [38] reported that, worldwide, the black carbon amount due to savanna fires could be in the range of 4–40 Tg C/year. However, about 10% of this goes in the form of aerosol into the atmosphere [37]. In the Northern Territory in 1992, these aerosols accounted for about 5.23 × 10^9^ g of particulate matter <2.5 µm in diameter [39]. Such emissions could potentially increase solar energy absorption [40] and thereby accelerate global warming. On the other hand, EDS prescribed burning can reduce emissions of black carbon aerosols by reducing the amount of carbon exposed to fire and thereby help to mitigate this impact. If this benefit is accounted for and credited to farmers with the savanna burning methodology, this could increase its value and encourage them to participate.

There is currently a lack of long-term soil, biomass and black carbon aerosol related experimental research in the non-tropical savanna areas of Australia. Omission of these sequestration and emission components is problematic and needs to be addressed both in the current northern savanna burning methodology and any future developments for other regions. This demands long-term experimental research for different vegetation, climatic and edaphic zones. In the past, the Australian Government has adopted a rigid 100 year permanence policy but is now taking a more flexible approach and accepting both 100- and 25-year permanence [17]. Therefore, the time frame of experiments for the development of scientifically robust methodology should be at least 25 years.

#### Inclusion of termite related CH_4_ emissions

Termites are wood-eating insects, globally abundant in tropical to temperate regions between 45°N and 45°S [41]. During the digestion of organic matter, they produce CH_4_ [42]. Annually, over 580 Tg of CH_4_ are released into the atmosphere [43] of which termites are estimated to contribute up to 19% [44]. Much of Australia lies within the 45°S latitude limit and supports numerous species of termites [41]. In Australia, CH_4_ flux estimates from termite mounds could be in the range of 0.2–1.6 Mt/year [45].

Research conducted by Jamali et al. [44] in the Northern Territory indicates that the quantity of CH_4_ emissions from termites largely depends on the species, time of day and season. CH_4_ flux is strongly positively correlated with mound temperature—lowest in the coolest time of the day (7 a.m.) and greatest at the warmest time of the day (3 p.m.)—probably due to accelerated methanogenesis processes in the termite gut. CH_4_ flux was also 5–26 times lower in the dry season than in the wet season [44]. Species of termites with larger hindguts may host more CH_4_-producing symbionts and produce more methane [46].

Early dry season (EDS) burning could change the dynamics of CH_4_ emissions from termites. Both EDS and LDS burning may temporarily reduce termite activity and lead to a net increase in soil CH_4_ uptake, as oxidation is no longer offset by termite emissions [47]. However, since the frequency of EDS burning is higher than LDS burning, EDS burning may have greater net CH_4_ benefits. This effect is attributable to anthropogenic management and may therefore be accounted for carbon credits. However, it is not yet known whether there is a significant change in termite activity post-EDS burning compared to that of the pre-EDS burning period. Also, it is not known whether EDS burning is better than LDS burning in the long-run in terms of net CH_4_ emissions.

There is currently insufficient evidence from research in these areas; hence, termite related CH_4_ emissions are not included in the current Australian Government savanna burning methodology and nor are they accounted for by the UNFCCC. If the CH_4_ benefits of EDS burning were greater than LDS burning and able to be accounted and included in the savanna burning methodology, it is likely that uptake of this method could increase. For this to happen, comprehensive research into the ecological function of local termite species and the impact of EDS burning on CH_4_ fluxes in different vegetation types and under a range of climatic conditions is crucial.

#### Inclusion of N_2_O emissions associated with microbial activity

N_2_O has 310 times more global warming potential than CO_2_ [13]. Changing fire regimes and additional unburnt biomass may alter soil N_2_O dynamics through changes in the microbial processes of nitrification and de-nitrification. Unburnt plant material could be a potential source of carbon and energy for heterotrophic denitrifying organisms which may enhance the rate of de-nitrification [48–51]. GHG emissions due to release by microorganisms occur over a longer period of time than is the case for burning; however, rates of emissions by micro-organisms largely depend on several climatic and edaphic factors. A methodology that accurately accounts for the GHG emissions due to microorganisms under different site specific conditions is very important.

Recent research in Australia reports that the rates of N_2_O emissions from savanna soils [27] and rangelands soils [52] are comparatively low. This may be due to low nitrogen availability and tight nitrogen cycling [53]. If so, there may be little difference in N_2_O emissions from prescribed burnt and unburnt soils in nitrogen poor areas. However, termite mounds have been identified as a significant point source of N_2_O [54]. However, an in vitro incubation study reported that the N_2_O production rates were higher in termites feeding on soil and fungi with higher nitrogen content, compared to those feeding on nitrogen poor wood [54]. Further research into N_2_O emissions associated with microbial activities in different soil and feed types (with different N levels) and under different climatic conditions is essential. Inclusion of microbial N_2_O emissions would enhance the credibility of the developed methodology.

#### Identify trade-offs between maintaining biodiversity and reducing GHG emissions

In Australia, both conserving biodiversity and reducing GHG emissions are equally important [55, 56]. Australia is one of seventeen countries described as being ‘megadiverse’ (http://www.biodiversitya-z.org/content/megadiverse-countries); in addition, it is also the highest per-capita GHG emitting country in the world (24 tCO_2_e/person) [57]. Several researchers suggest that there is a complex relationship between biodiversity and carbon sequestration benefits [58–60], but ways of achieving synergies between these two benefits with improved fire management are little researched. Altered fire regimes such as long-term fire exclusion can change vegetation patterns and lead to biodiversity decline [61–63]. At some point, it is likely that the trade-off between maintaining biodiversity values and increasing GHG abatement will be minimised. Richards et al. [28] and Williams et al. [5] suggest that it is crucial to determine the stage at which these two benefits can be optimised, especially in biodiversity hotspot areas. This would help us identify thresholds and optimise both outcomes.

#### Determine whether the currently adopted baseline (based on 10 years of data in high rainfall region and 15 year of data in low rainfall region) is adequate for other parts of Australia

Australia spans a latitudinal range from 10°S in the tropical north to 44°S in the south. From north to south the seasonality of rainfall changes, inter-annual rainfall variability becomes more extreme, grass and shrub vegetation becomes more dominant, and fire intervals increase [64, 65]. East–west rainfall gradients also exist with mean annual rainfall diminishing from >1000 mm in coastal regions to <400 mm some 1000 km inland. In some parts, such as the non-spinifex grasslands of south–eastern Northern Territory and south-western Queensland, fire only occurs following periods of above-average rainfall for two or three consecutive seasons [65]. Therefore, the 10- and 15-year baseline periods adopted in the current methodology for high rainfall areas (average annual rainfall of >1000 mm) and low rainfall area average annual rainfall of 600–1000 mm rainfall), respectively, in Northern Australia may not be applicable to other parts of Australia. The 10-year higher rainfall baseline period covers approximately three fire cycles and provides a reliable foundation for estimating the emissions from project areas [17]. However, the 15-year baseline period for lower rainfall regions will cover only one or two fire cycles [17] and may not provide enough data for baseline estimation. Similarly, in other parts of Australia where annual rainfall averages less than 600 mm and fire intervals are longer, a more extended baseline period would be required. Moreover, in these areas, there is a limited satellite record available for determining historic fire regimes. Further research is needed to enable accurate estimation of emissions over appropriate baseline periods for other parts of Australia.

#### The concept of “leverage” in quantifying the risk of a savanna burning project

Recently, the concept of ‘leverage’ [66]—the reduction in area burnt by unplanned fire per unit area treated with planned fire—has been developed to evaluate the effectiveness of prescribed burning. In the tropical Australian savanna, the leverage value is >1 (i.e. prescribed burning treatment leads to a reduction in the total area burnt) [67], while in South Eastern Australian forests it is less <1 [6]. There are two main reasons for a higher leverage in tropical savanna areas compared to that in southern areas: (1) higher initial rates of fuel accumulation; and (2) higher fire frequencies in northern savanna areas than in other parts of Australia [6]. The lower leverage value for temperate forests indicates that risk reduction in these regions is more difficult to achieve than in the northern savanna area. A modelling result suggested that the use of EDS burning in temperate forests may not yield a net reduction in carbon emissions [68]. Similar analysis for sub-tropical parts of Australia could aid decisions about whether to develop such a methodology as lower leverage may discourage landholder participation.

#### Understanding the complex interactions between fire regimes and a changing climate regime

It is likely that climate change will impact fire regimes through changes in weather related attributes (temperature, rainfall, humidity, wind and radiation), fuel related attributes (changes in moisture content on vegetation and fuels), and carbon fertilisation [5]. In Australia, the impact of fuel on fire incidence diminishes from north to south while weather becomes a more prominent driver [6]. Therefore, climate change and climate variability could play an increasingly major role in the fire regimes in southern parts of Australia. Comprehensive research into the complex interactions between fire regimes and changing climatic regimes is crucial for identifying those regions and ecosystems likely to be most fire prone in the future. Regions which would currently not be cost-effective for fire management projects under the savanna burning methodology may become more beneficial over time due to changing fire risk under future climatic conditions.

#### Understanding the implications of large wildfires for avoided emissions

Even with careful management, there is always the possibility that a large wildfire may burn significant areas of savanna. As a result, there is ongoing debate about whether such a fire could wipe out gains in avoided emissions achieved during the unburned period. Satellite imagery and analysis of results from the field show that there is only minor impact on the overall GHG emissions in northern savannas if large wildfires are infrequent (http://savanna.cdu.edu.au/). The northern savannas do not accumulate large amounts of fuel as biomass is rapidly decomposed by microorganisms, fungi and termites [69] and the amount of available fuel for burning tends to level out some 2–3 years post-fire [69].

However, in southern regions, fuel is less rapidly decomposed by microorganisms and may accumulate over longer periods in the absence of prescribed burning. Therefore, if a wildfire occurs, there is a greater chance of significant GHG emissions. The Australian Government’s savanna burning methodology, which is currently based on northern Australian fire management practices, is not applicable to southern parts and a methodology which considers the possibility of large wildfires is essential. The economics of this issue also need to be analysed as there are significant implications in terms of the relative costs and benefits of prescribed burning in southern forests.

#### Conducting a comprehensive cost benefit analysis of the need

A comprehensive cost benefit analysis of developing a robust savanna burning methodology for other parts of Australia is needed for three reasons. Firstly, as noted, in a given time the amount of burnable fuel load in southern parts of Australia may be relatively low. Even if the fuel load is high, fire frequency could be low due to climatic factors. Therefore, unlike in Northern Australia, an extensive annual prescribed burning program may not be necessary in many parts of southern Australia.

Secondly, the cost of savanna burning is location specific and depends on the costs associated with collaboration with partner organisations and provision of equipment and infrastructure (out-stations, access roads etc.) and fire frequency. Northern Australia has well-developed infrastructure as prescribed burning has been undertaken there for many years [70]. A current savanna burning project—the Western Arnhem Land Fire Abatement (WALFA) in Northern Territory, covering an area of 28,000 km^2^ of the Arnhem Plateau adjoining Kakadu and Nitmiluk National Parks—provides an indication of fire abatement costs. After seven years of implementation, the project has reduced emissions of CH_4_ and N_2_O by 37.7%, relative to the pre-determined baseline [71]. The project delivered a mean annual abatement of 141,400 t CO_2_-e over the period 2005–2010 at an estimated annual cost of $1.75 M [72]. This amounts to $12.4/tCO_2_-e abated. This was an attractive price when the Australia Government carbon price was $23/tCO_2_-e. However, with the scrapping of the carbon tax by the current Government, there is currently very little demand for carbon credits in Australia and the average carbon price at the recent auction was just $10.23 [73]. However, this project will continue to be more attractive than afforestation, reforestation and forest management projects and comparable to soil and livestock management projects [72, 74]. Moreover, this type of project provides employment opportunities and financial resources for natural resource management and enables traditional land owners to restore and refine their management practices [70].

Thirdly, in southern parts of Australia where fuel loads and the frequency of fire are often low, costs may outweigh revenues where only Kyoto eligible carbon credits (CH_4_ and N_2_O abatement) are considered. If all benefits, including biosequestration and other co-benefits (such as social, biodiversity etc.), are considered, revenue could outweigh costs. Therefore, a comprehensive cost benefit analysis including all tangible and intangible benefits for all vegetation types is strongly recommended. This analysis would provide a sound basis for decision-making for both policy makers (on whether to develop such methodology) and for landholders (on whether to participate in a savanna burning project on their property).

#### Estimating fuel loads, fuel components (size classes), fire severity class, burning efficiency and emissions factors for CH_4_ and N_2_O in different vegetation types

A range of issues would need to be addressed if the Australian Government decided to develop prescribed burning methodologies for other parts of the country. In the current northern Australia methodology, as noted, emissions are determined by patchiness, fire severity class, burning efficiency, fuel loads, emissions factors, carbon content and the nitrogen to carbon ratio. These parameters and their values are based on 10–20 years of research in Northern Australia and vary by vegetation fuel type, fuel components (size classes), fire season, fire interval and climatic condition [65, 75–78].

Significant differences in climatic, edaphic and topographic factors, as well as vegetation type, are found in other parts of the country, meaning that parameter values could differ significantly from those in northern Australia. Similarly, forest patch size also significantly influences soil and biomass carbon sequestration after fire [79]. Therefore, if a prescribed burning methodology is to be developed for southern Australia, as part of Australia’s commitment to reducing GHG emissions, significant effort will be required in order to establish the necessary criteria to enable this to occur.

## Conclusions

Savannas occupy about one-sixth of global land area and is maintained by fire, yet fire is also a major source of global emissions. If savanna fire is managed properly, a large portion of these emissions can be avoided. Several domestic and international voluntary and mandatory emissions reduction approaches are in place to reward emissions reduction activities. The Australian government is at the forefront in this area and has developed a credible savanna burning methodology for tropical parts of Australia which has been taken up by landowners in order to receive Australian Carbon Credit Units under the Australian Government’s Emission Reduction Fund.

This review provides new insights and understanding of the current savanna burning methodology and the issues associated with extending it to other savanna landscapes across Australia. Firstly, the development of a similar methodology for savanna burning in the subtropical and temperate regions of Australia will need to identify the critical characteristics of savanna vegetation types (e.g. historical fire regime, fuel components, fuel accumulation, burning efficiencies, emissions factors for CH_4_ and N_2_O) and focus on details of the prescribed burning fire regime (timing, frequency and intensity of burning) within each savanna vegetation type. Secondly, the dynamics of organisms such as termites and nitrifying/denitrifying bacteria and their roles in carbon sequestration (in soils and biomass) and GHG emissions in these ecosystems will also need to be established and quantified. Lastly, a baseline reference period will need to be established which accommodates the historical variability of fire occurrence in these vegetation types to enable measurement of gains and losses under the methodology.

This paper presents a critical review of these factors and provides a template for ongoing discussion around the feasibility of developing a savanna burning methodology for other parts of Australia to assist in quantifying Australia’s contributions to GHG emissions reduction and climate change abatement. The paper also informs policy development in other countries that are intent on developing similar emissions reduction strategies.
